# Cough as a Clue: Tracheal Endocarcinoma Unveiled

**DOI:** 10.7759/cureus.40335

**Published:** 2023-06-12

**Authors:** Hajar Charii, Asmae Boudouh, Oumayma Mezouari, Othman Moueqqit, Hatim Kouismi

**Affiliations:** 1 Department of Respiratory Diseases, Mohammed VI University Hospital, Oujda, MAR; 2 Department of Respiratory Diseases, Faculty of Medicine and Pharmacy, Mohammed First University, Oujda, MAR; 3 General Medicine, Faculty of Medicine and Pharmacy, Mohammed First University, Oujda, MAR

**Keywords:** tracheal tumor, chronic cough, diagnosis of asthma, inspiratory dyspnea, tracheal adenocarcinoma

## Abstract

Tracheal adenocarcinoma (TAC) is a rare malignancy often characterized by significant delays in diagnosis, often attributed to the non-specific nature of symptoms, leading to subsequent challenges in management. The prognosis remains poor, highlighting the need for early detection and multidisciplinary treatment strategies. Surgical resection is recommended for eligible patients, followed by postsurgical irradiation. However, further research is required to give a better perspective on therapeutic interventions and enhance patient outcomes.

This paper reports the case of a 50-year-old male, who presented with dyspnea, hemoptysis, and cough. The computed tomography (CT) revealed an intratracheal tissue mass. The cytological examination and immunocytochemistry confirmed the diagnosis of primary adenocarcinoma in the trachea. The treatment involved silicone tracheobronchial Y-stent followed by adjuvant chemotherapy with carboplatin and paclitaxel, and radiotherapy (60 Gray) with good clinical improvement.

## Introduction

Tracheal adenocarcinoma (TAC) is an extremely uncommon and poorly reported tumor accounting for less than 1% of all malignancies [1]. The diagnosis is often made at an advanced stage, primarily attributed to the delayed onset of nonspecific symptoms such as hemoptysis, dyspnea, cough, and stridor [1]. Optimal management strategies hinge upon a careful assessment of the tumor stage and the feasibility of the surgical intervention, while radiation therapy and chemotherapy serve as viable alternative options [1,2]. Due to the limited clinical data, a multidisciplinary approach is indispensable for comprehensive patient care. It is imperative to conduct further investigations to enhance our understanding and to devise more efficacious therapeutic modalities for this condition.

## Case presentation

A 50-year-old male with a smoking history of 35 pack-years presented with an acute worsening of the frequency and the severity of cough superimposing a chronic dyspnea that persisted for eight months along with hemoptysis, asthenia, and significant weight loss. In the early stages of his presentation, the patient's symptoms of persistent cough and wheezing dyspnea were initially attributed to asthma and managed accordingly.

**Figure 1:**
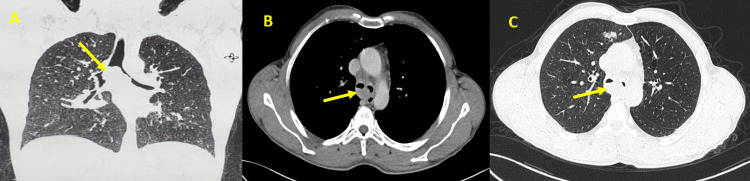
CT scan images CT scan reveals a luminal narrowing due to intratracheal tissue mass (arrows). Figure A shows the coronal view with a parenchymal window, Figure B displays the axial view with a mediastinal window, and Figure C presents the axial view with a parenchymal window.

Physical examination revealed wheezing and stridor. The patient exhibited an oxygen saturation of 88% on room air. Chest radiography showed no abnormalities, but a subsequent chest computed tomography (CT) scan revealed a 24*29 mm intratracheal tissue mass accompanied by bronchial dilatation in the middle lobe (Figure 1).

**Figure 2:**
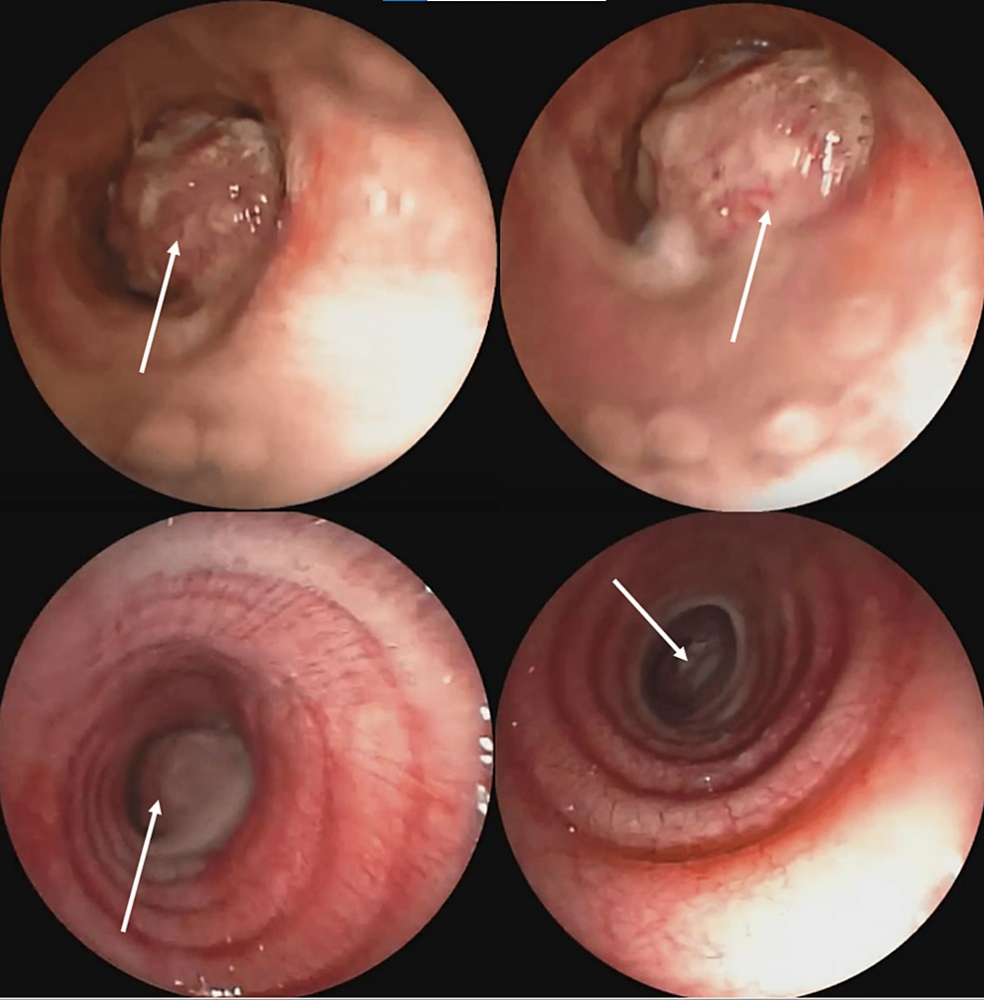
Bronchoscopic view of the tumor The tumor is causing tracheal obstruction and infiltration, with significant luminal occupation. Rigid bronchoscopy confirms tumor presence proximal to the carina (arrows), obstructing the main bronchi.

During flexible bronchoscopy, a white tumor with a propensity to bleed was observed (Figure 2). The tumor obstructed and infiltrated the tracheal wall in the lower 1/3 of the trachea, occupying more than 70% of the tracheal lumen. Rigid bronchoscopy confirmed the presence of a tumoral mass located proximal to the carina obstructing the bilateral bronchial tree and around 70-80% of the tracheal lumen.

**Figure 3:**
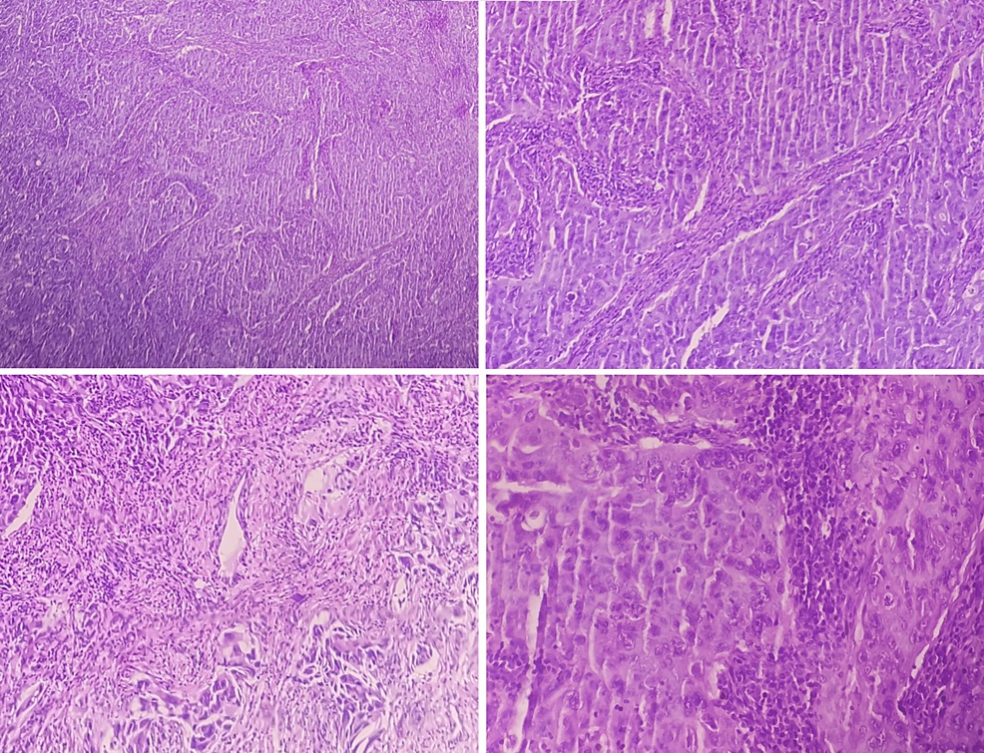
Histopathological images Histopathological images demonstrating poorly differentiated invasive squamous cell carcinoma with features suggestive of invasive solid adenocarcinoma.

Histopathological evaluation of the biopsy sample revealed a poorly differentiated invasive squamous cell carcinoma, with immunocytochemistry favoring an invasive solid adenocarcinoma (Figure 3). Further staging investigations revealed the presence of a locally advanced subcarinal mediastinal tumor, hilar lymph node involvement, and a pulmonary nodule in the right upper lobe. The tumor was classified as T4N1M1.

**Figure 4:**
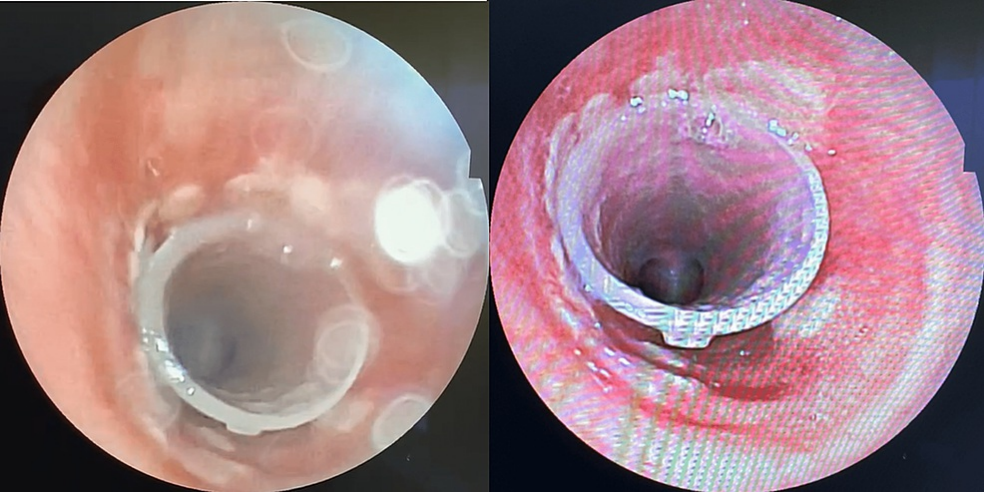
Endobronchial imaging Endobronchial imaging showing the placement of the silicone tracheobronchial Y-stent: Relief of tracheal obstruction visualized

Following a multidisciplinary consultation, the decision was made to proceed with four sessions of chemotherapy with carboplatin and paclitaxel and radiotherapy (60 Gray) after placing a silicone tracheobronchial Y-stent (limb of 16 mm diameter and 4 cm length) to alleviate tracheal obstruction and dilatation after removing part of the tumor (Figure 4). The patient was subsequently transferred to the thoracic surgery team, which successfully relieved the tracheal obstruction and dilatation.

Significant clinical improvement was noted immediately afterward, with the resolution of respiratory symptoms. A silicone tracheobronchial Y-stent placement with the same dimensions was scheduled for future management. Unfortunately, five months later, the patient died from confirmed coronavirus disease 2019 (COVID-19) pneumonia.

## Discussion

Tracheal cancers are extremely rare, with an estimated incidence of approximately 0.1 per 100,000 individuals annually [3]. To date, the largest analysis of primary tracheal tumors was conducted using the comprehensive Surveillance, Epidemiology, and End Results (SEER) database, which included a total of 578 cases [3]. Within this report, 55% were male, and the prevailing histological type identified was squamous cell carcinoma, accounting for 45% of the cases [3]. Other histological types observed encompassed adenoid cystic carcinoma, small cell carcinoma, large cell carcinoma, sarcoma, adenocarcinoma, and unspecified or undifferentiated carcinoma. Notably, while primary tracheal tumors in adults are predominantly malignant, representing nearly 90% of cases, the rate of malignancy in children is significantly lower, ranging from 10 to 30% of cases [3].

TAC rarity and intricate management give rise to notable diagnostic and therapeutic complexities [1]. First, tracheal tumors often mimic asthma during the initial stages and the diagnosis is frequently delayed due to the late emergence of nonspecific symptoms such as hemoptysis, dyspnea, coughing, and stridor. While rare, the involvement of adjacent structures in TAC can lead to additional symptoms such as dysphagia and hoarseness [4]. The misdiagnosis is further supported by the presentation of a normal radiograph, adding to the challenge of accurate identification [1]. In our particular case, the patient manifested similar presentations associated with weight loss and asthenia and followed by a normal chest radiograph which are typical of the clinical features reported in TAC.

Chest radiographs rarely detect changes indicative of tracheal neoplasms [4]. This is primarily due to the superimposition of the thoracic spine and mediastinal structures on the trachea in the posteroanterior view. Therefore, the lateral view of a chest X-ray is often more helpful in aiding in diagnosing tracheal neoplasms [4]. However, physicians should be aware that chest radiographs may initially appear normal in most patients with tracheal neoplasms.

The diagnosis of TAC in our patient was confirmed through evaluation using flexible and rigid bronchoscopy, along with histopathological examination. The presence of a white growing tumor observed during flexible bronchoscopy, followed by a biopsy, revealed a poorly differentiated invasive squamous cell carcinoma. Immunocytochemistry results further supported the presence of invasive solid adenocarcinoma, highlighting the heterogeneity commonly observed in tracheal tumors [5].

The prognosis for patients with malignant tumors of the trachea is generally poor, and the long-term median survival of patients with TAC undergoing postoperative irradiation therapy is still poorly explored [2]. However, the prognosis for patients who are eligible for surgical resection is generally more favorable compared to those who are not [2]. Therefore, surgical resection is often recommended as the primary treatment approach for most cases of primary tracheal tumors, regardless of tumor burden, margin status, histology, or nodal status. All patients who undergo surgical resection for tracheal neoplasms require postsurgical irradiation as part of their treatment protocol [6].

Overall, early recognition, accurate diagnosis, and comprehensive treatment planning involving multidisciplinary approaches are crucial for optimizing patient outcomes. Further research is needed to better understand the biology of TAC and develop targeted therapies to improve patient prognosis.

## Conclusions

TAC represents a rare and challenging form of cancer. The limited prevalence of this malignancy underscores the difficulties in its diagnosis and treatment. A multidisciplinary approach is essential to address complex management effectively. Furthermore, ongoing research endeavors are crucial to expand our knowledge, refine diagnostic techniques, and establish optimal therapeutic approaches for this rare condition.
